# Risk Factors and Clinical Significance of Urologic Injury in Cesarean Hysterectomy for Placenta Accreta Spectrum

**DOI:** 10.3390/jcm14207199

**Published:** 2025-10-13

**Authors:** J. Connor Mulhall, Kayla E. Ireland, John J. Byrne, Patrick S. Ramsey, Georgia A. McCann, Jessian L. Munoz

**Affiliations:** 1 Department of Obstetrics and Gynecology, Division of Maternal Fetal Medicine, Division of Fetal Intervention, Baylor College of Medicine, Texas Children’s Hospital, Houston, TX 77030, USA; joseph.mulhall@bcm.edu; 2 Department of Obstetrics & Gynecology, University of Texas Health Sciences Center at San Antonio, San Antonio, TX 78229, USA; ireland@uthscsa.edu (K.E.I.); byrnej@uthscsa.edu (J.J.B.); ramseyp@uthscsa.edu (P.S.R.); mccanng@uthscsa.edu (G.A.M.)

**Keywords:** placenta accreta spectrum, urologic injury, cesarean hysterectomy, antepartum vaginal bleeding, ultrasound

## Abstract

**Background/Objectives**: Placenta accreta spectrum (PAS) is an obstetric condition with placental adherence to the underling myometrium characterized by significant surgical morbidity at time of delivery. PAS delivery is most commonly performed by cesarean hysterectomy. The most common morbidities associated with PAS cesarean hysterectomy are blood transfusion, intensive care unit admission and urinary tract injuries. This requires interdisciplinary team management including obstetricians and urologists. Our objective was to identify pre- and intra-operative risk factors for urologic injury in this high-risk condition. **Methods**: A retrospective cohort study was performed at a single tertiary center with the Center for the Management of Placenta Accreta Spectrum disorders from 2012 to 2022. Urologic injuries were considered as injury to either the bladder or ureters. Furthermore, bladder injuries were subdivided into those inherent to the procedure (intentional cystotomy) and those considered unplanned complications (incidental cystotomy). Inclusion criteria required complete antenatal documentation for assessment, and these were accessed by electronic medical records. Multivariate analysis was performed for significant variables on univariate analysis. **Results**: During the 11-year study period, 146 cases of PAS were managed by our team. Of these, 39 (26.7%) were complicated by urologic injury. Intentional cystotomies were performed in 28.2% (11/39) of cases. There were 28 (28/39, 71.8%) incidental cystotomies and 5 (5/39, 12.8%) ureteral injuries were encountered. Of note, all 5 patients with ureteral injuries also had cystotomies. Upon univariate analysis, anterior placentation (OR 2.96 [1.94, 4.67], *p* = 0.04), percreta by antenatal ultrasound (OR 2.59 [1.13, 5.9], *p* = 0.02) and >2 pre-delivery vaginal bleeding episodes (OR 4.27 [1.54, 12.16], *p* = 0.005) were associated with urologic injury. Multivariate analysis revealed the independent significance of these variables. Of note, the presence of zero, one, two, and all three risk factors were associated with urologic injury rates of 11.1%, 22.5%, 41.9%, and 71.4%, respectively. **Conclusions**: Urologic injury during cesarean hysterectomy occurs in almost one-third of cases. Pre-operative risk factors may be used to identify those at greater risk for this surgical complication. Determining patient risk allows for the use of resources such as formal urology consultation, surgical planning, and intraoperative assistance, as well as detailed patient counseling.

## 1. Introduction

Placenta accreta spectrum (PAS) is a complex obstetric condition with varying degrees of placental adherence to uterine myometrium. PAS has been classically stratified by depth of placental invasion through the myometrium (accreta, increta, and percreta). Those three subtypes can be determined by pathological or clinical assessment of invasion, with accreta referring to placental adherence to the myometrium, increta if the myometrium is invaded, and percreta when the depth of invasion extends to the outer uterine serosa [1,2]. The current detection of PAS is dependent on clinical suspicion and consideration of patient-specific historical risk factors. The primary risk factors for PAS are the presence of a placenta previa (where the placenta covers the internal cervical os) and a history of cesarean delivery in a prior pregnancy [3]. A direct relationship is observed between the number of prior cesarean deliveries and PAS risk. As global cesarean section rates continue to increase, so does the incidence of PAS [3,4]. This is displayed by the prevalence of PAS in the United States, which rose more than 6-fold from 1982 to 2002 to a rate of 0.2% [4].

Pregnancies complicated by PAS carry risks for significant maternal adverse outcomes. The atypical placental attachment prohibits the normal separation and expulsion of the placenta, thus inhibiting subsequent uterine contraction and involution. During the gravid state, maternal blood flow through the uterine–placental interface is approximately 20% of cardiac output, or 500 mL/min. Thus, bleeding at this interface results in large volume blood loss and significant maternal morbidity. This leads to increased rates of postpartum hemorrhage, which can be life-threatening and require a blood transfusion. Additionally, due to the depth of placental invasion and obliteration of standard surgical planes, there is an increased risk for intraoperative organ injury, particularly of the genitourinary (GU) system [5,6]. These cases require a multi-disciplinary approach, often including maternal–fetal medicine, neonatology, anesthesiology, urology, and gynecologic oncology [7]. Given the highly coordinated nature of these cases, it is recommended that delivery occur prior to the onset of labor to allow for adequate preparation by all appropriate teams [8].

It has been well described that the GU system is the most frequent site of surgical injury during cesarean hysterectomy for PAS. A 2022 systematic review and meta-analysis of 56 studies showed a urologic complication rate of 19.4% in this patient population. Cystotomy, both intentional and unintentional, was the most common urologic injury identified, followed by ureteral injury, then fistula development [9]. Depth of invasion, specifically placenta percreta, has been cited as an associated intraoperative factor for GU injury [10,11,12].

Though the frequency and type of GU injuries have been well described in prior studies, clinical factors associated with GU injury have not been as well delineated. In this study, we aim to identify preoperative factors predictive of GU injury during cesarean hysterectomy for PAS. We also detail differences in perioperative complications between patients who sustain GU injuries.

## 2. Materials and Methods

This is a retrospective cohort study of patients who presented to the University of Texas Health San Antonio and University Hospital System for PAS management between 2012 and 2022. Institutional review board (IRB) approval was obtained prior to collecting patient information from the electronic medical records. Inclusion criteria included viable pregnancy, maternal age between 18 and 55 years, antenatal suspicion for PAS (based on historical risk factors, ultrasound, or MRI findings), and histopathological confirmation/characterization of PAS by a board-certified pathologist. Patients were excluded if they delivered at a different institution, delivered at <20 weeks gestational age, or had incomplete medical records.

All patients were managed with cesarean hysterectomy. At this center, the PAS team includes a multidisciplinary group of subspecialists, including maternal–fetal medicine, urology, gynecologic oncology, obstetric anesthesia, interventional radiology, transfusion medicine, and trauma surgery. Ureteral stents were planned for all cases when they were clinically feasible and safe. In cases of suspected placenta percreta, uterine artery embolization (UAE) was performed following delivery of the neonate before proceeding for hysterectomy.

Genitourinary injury was defined as either cystotomy or ureteral injury. The incidence of cystotomy was subdivided into either intentional or incidental cystotomy. Aspects of maternal baseline conditions, ultrasound assessment, pregnancy complications, and operative characteristics were obtained from the electronic medical record. Antenatal PAS diagnosis was made on the bases of ultrasound and/or MRI findings, and the final PAS diagnosis was determined by tissue evaluation by a faculty pathologist.

Research Electronic Data Capture (REDCap) software (Version 15.5.15) hosted by the University of Texas Health San Antonio was used for data collection and storage. REDCap (Research Electronic Data Capture, Vanderbilt University, Nashville, TN, USA) is a secure, web-based application designed to support data capture for research studies. Investigators extracted data from the electronic medical record and manually entered into REDCap.

Normal distribution was determined by a Shapiro–Wilk test result greater than 0.05. Pearson’s chi-square (χ2), Fisher’s exact tests, the Mann–Whitney U test, and *T*-tests were applied when appropriate. Categorical factors were summarized using frequencies and percentages, while continuous measure summaries used means ± SD or median and range as appropriate; *p*-values < 0.05 were considered significant for a two-tailed analysis. Univariate and multivariate logistical regression was performed to assess the impact of individual risk factors and GU injury. The significant risk factors from the univariate analysis (anterior placenta percreta by US and vaginal bleeding > 2) were included in this analysis to confirm independent significance. Statistical analysis was performed using Graphpad software (version 10.0.2).

## 3. Results

During the 11-year study period, 146 cases of PAS were managed by our team. Among this cohort, 39 cases (26.7%) were complicated by urologic injury. Intentional cystotomy was performed in 28.2% (11/39) of cases. Incidental cystotomy occurred in 71.8% (28/39) of cases, and 5 (5/39, 12.8%) ureteral injuries occurred. Of note, all cases of ureteral injuries also had cystotomies.

The baseline demographics of the study population are outlined in Table 1. The patients in our cohort are divided into two groups, patients without GU injury and patients with GU injury, respectively. Patients who sustained GU injury were more likely to have higher number of prior cesarean deliveries (CD), anterior placentation, and ultrasound findings suggesting placenta percreta. Other demographic factors were similar between the groups, including age, BMI, and gestational age at time of delivery.

Table 2 summarizes the antepartum complications that occurred within the cohort. Overall, this population had a high rate of antepartum admission, and this rate was not statistically different between groups. Patients who sustained GU injury were significantly more likely to have greater than 2 episodes of antepartum vaginal bleeding compared to those who did not have a GU injury. There were no other differences in other antepartum complications between the two groups.

In regard to delivery and operative outcomes (Table 3), both groups received preoperative uterine artery embolization (UAE) and prophylactic ureteral stent placement at similar rates. Cases complicated by GU injury had significantly longer operative times and estimated blood loss (EBL). Patients who had GU injuries were more likely to require intensive care unit (ICU) admission and had longer postoperative lengths of stay (LOS).

**Figure 1:**
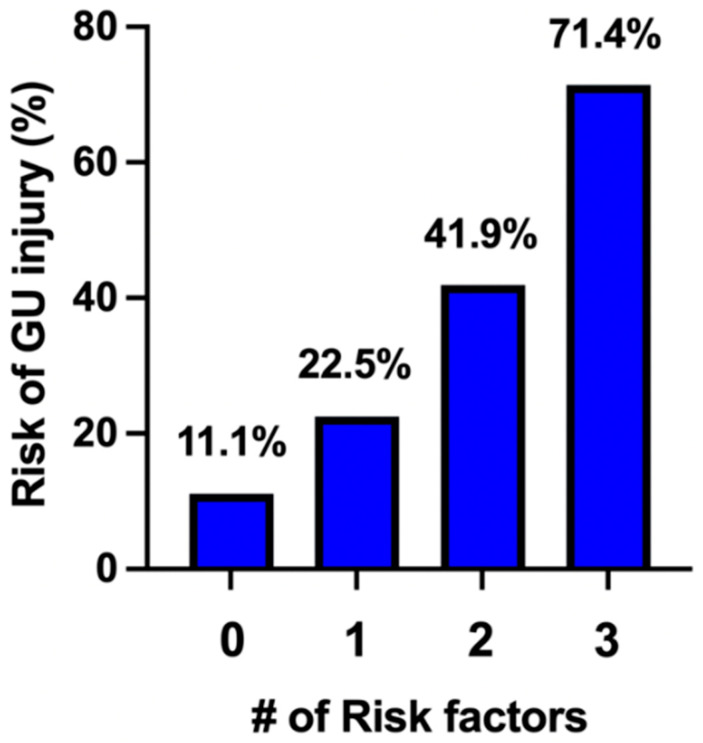
Risk determination of GU injury.

The significant antepartum findings associated with GU injury, as noted above, were evaluated by univariate analysis. Table 4 presents the three factors significantly associated with GU injury, which include anterior placentation, suspected percreta by ultrasonographic assessment, and greater than two vaginal bleeding episodes during pregnancy. These three factors were found to have independent significance by multivariate analysis. Figure 1 demonstrates the additive risk associated with each successive factor for GU injury during PAS surgery. For patients with no identified risk factor, there was an 11.1% rate of GU injury. For patients with one, two, and three risk factors, there was a 22.5%, 41.9%, and 71.4% rate of GU injury, respectively.

## 4. Discussion

The findings of this study identified anterior placentation, suspected placenta percreta, and >2 episodes of antepartum vaginal bleeding as predictive of GU injury during cesarean hysterectomy for PAS. Through multivariate analysis, we show that these preoperative factors have independent significance for the prediction of GU injury. These factors have an additive effect for the risk of GU injury. Patients who had GU injury were more likely to have other adverse surgical outcomes, including increased blood loss, operative time, ICU admission rates, and postoperative lengths of stay.

Our cohort had a 26.7% rate of GU injury, the majority of those being cystotomies. This is similar to other studies reporting rates of GU injury during PAS surgery. Lucidi et al. recently published a systematic review and meta-analysis of 62 studies reporting on the incidence and type of GU injury during PAS surgery and further separated outcomes by type of delivery intervention and final PAS grade by histopathologic evaluation. For patients who underwent hysterectomy at the time of delivery for PAS, as in our cohort, they reported an incidence of 19.4% (95% CI 16.3–22.6). In the subset of patients with histopathologic diagnosis of placenta percreta, the rate of GU injury was 37.5% (95% CI 30.6–44.7) [9]. More than half of the patients in our cohort had placenta percreta confirmed by pathology, so seemingly our rate of GU injury is in line with previously published literature.

There have been few prior studies evaluating preoperative risk factors associated with GU injury in this patient population. Friedrich et al. similarly identified suspicion for placenta percreta as a risk factor for GU injury, as well as a number of previous cesarean deliveries. Notably, their cohort had a relatively low rate of GU complication (9.3%, 29/312). Additionally, there are significant practice pattern differences geographically, as only 14.4% of their cohort underwent hysterectomy compared to 100% in our cohort [11]. Variation in surgical technique likely influences the difference in GU injury.

A recent retrospective study by Hage et al. also reported on increased risk for GU injury in patients with multiple prior uterine surgeries, in addition to a higher number of cesarean deliveries. Their cohort had a rate of GU injury of 21.5%, the majority of which were cystotomy. They detail that 75% of cystotomies occurred in the dome or posterior wall of the bladder, with injury to the trigone, lateral, or anterior bladder walls occurring less frequently [12].

Our study found that antepartum vaginal bleeding, specifically, greater than 2 bleeding episodes, was significantly associated with intraoperative GU injury. The association of vaginal bleeding episodes with other adverse events in cases of PAS has been previously described. There is an association between antenatal vaginal bleeding and an earlier gestational age at delivery, as well as adverse maternal composite outcomes including EBL ≥ 2 L, transfusion ≥ 4 units, ICU admission, and postoperative length of stay [8,13]. We also show that, in addition to ultrasound factors showing suspected placenta percreta and/or anterior placentation, patients with >2 episodes of vaginal bleeding during pregnancy are at an additive risk for GU injury than if relying on ultrasonographic findings alone.

The presence and combination of these risk factors has significant implications during the antenatal assessment. Anterior placentation is more likely to involve larger portions of the prior hysterotomy when compared to posterior placentas, which may contain a smaller anterior portion to the prior uterine scars. In combination with suspected percretas, extensive dissection of the uterine–bladder interface is anticipated, which is likely the cause of GU injury in this cohort.

Strategies to prevent GU injuries in cases of PAS remain an area of ongoing investigation. Prophylactic ureteral stent placement has been proposed to reduce the incidence of GU injuries, with mixed results. While some individual studies report a reduction in GU injury with the use of ureteral stents, a recent systematic review and meta-analysis of 9 studies, including 848 patients showed no difference in GU complication rate [14]. It is notable that this analysis did not separate GU injury by location of injury along the GU tract. While it has been suggested that ureteral stent placement can assist with identifying the lateral and inferior aspects of the bladder and thus could potentially reduce cystotomy rates, as the majority of cystotomies occur at the bladder dome or posterior wall, this may not reduce the most common types of GU injury during PAS surgery [12,14,15].

Conservative management via delayed hysterectomy or partial uterine resection has also been proposed to reduce morbidity, including GU injury. While not always feasible, conservative management has been shown to be associated with reduced blood loss and transfusion rate [16]. Some studies have also reported on reduction of GU injury, though notably these are with relatively small cohorts, and GU injuries were not the primary outcome of the studies [17]. Further research and systematic reviews are necessary to determine if conservative management reduces the incidence of GU injuries in PAS cases.

Here we present a novel preoperative risk factor for GU injury during cesarean hysterectomy for PAS, as well as affirm that suspected placenta percreta is associated with GU injury. The limitations of our study include its retrospective nature, and so we are unable to prove causality of our findings. The patients presented in this cohort come from a single institution, which may limit its generalizability. In addition, as previously described, our center has a series of protocols for PAS management that may vary from those of other institutions. Lastly, additional prenatal and pathologic categories of PAS have been reported; these were described in recent years, and our study extends to years preceding these systems, thus they could not be included in our analysis.

The strengths of this study include its relatively large sample size for a rare obstetric condition. Also, all cases were managed by the same interdisciplinary team which employed protocolized approaches to PAS care, thus reducing variability among case management. The use and practice of protocols also allowed for implementation in emergent situations. Additionally, we only included cases where PAS was confirmed by pathologic diagnosis, not only on antenatal ultrasound findings. This approach was optimal given the variability in antenatal detection by imaging. Prospective studies from multiple centers are necessary to determine the validity and generalizability of the findings we present here.

The importance of preoperative risk assessment cannot be understated in cases of PAS. It has been shown that patients who are delivered at medical centers with multidisciplinary care teams with experience managing PAS have significantly improved outcomes [18,19]. Urologic expertise is a critical piece of this care team. Placement of ureteral stents, assistance with vesicouterine pouch dissection, and identification and repair of GU complications are all needed to reduce morbidity. This coordinated care is dependent on maintaining a high level of suspicion for the condition, including knowledge of risk factors for PAS and the associated morbidity and mortality associated with this condition.

## Figures and Tables

**Table 1:** Patient characteristics.

Characteristic	No GU Injury (n = 107)	GU Injury (n = 39)	*p*-Value
Age (years)	31.2 ± 5.5	31.5 ± 5.2	0.79
BMI (kg/m^2^)	33.1 ± 6.2	31.7 ± 5.7	0.22
Gravidity	4 [3, 6]	5 [4, 6]	0.19
Parity	3 [2, 3]	3 [2, 4]	0.02
History of C/S	96 (90)	39 (100)	0.04
Number of prior C/S	2 [1, 3]	3 [2, 4]	0.01
Tertiary referral	80 (74.8)	29 (74.4)	1.0
EGA at delivery	34 [33, 36]	34 [32, 35]	0.20
Placental location			
Anterior	79 (73.8)	35 (89.7)	0.04
Posterior	22 (18.7)	2 (5.1)	0.02
Lateral	6 (5.6)	2 (5.1)	1.0
PAS by Ultrasound			
Previa	29 (27.1)	9 (23.1)	0.68
Accreta	57 (53.2)	15 (38.5)	0.13
Increta	2 (1.9)	1 (2.6)	1.0
Percreta	19 (17.8)	14 (35.9)	0.03
Pregestational diabetes	8 (7.5)	3 (7.7)	1.0
Chronic Hypertension	12 (11.2)	2 (5.1)	0.35
Emergent delivery	29 (27.1)	15 (38.5)	0.22
Public Insurance	83 (77.7)	29 (74.4)	0.66

Values presented as mean ± SD, median [P25, P75], or N (column %), BMI = body mass index, C/S = cesarean section, EGA = estimated gestational age.

**Table 2:** Antepartum complications.

Complication	No GU Injury (n = 107)	GU Injury (n = 39)	*p*-Value
Antepartum admission	75 (70.1)	24 (61.5)	0.32
Antepartum LOS	1 [0, 3]	2 [0, 11]	0.70
PPROM	5 (4.7)	2 (5.1)	1.0
No. Vaginal bleeding			
X1	21 (19.6)	4 (10.3)	0.22
X2	16 (14.9)	4 (10.3)	0.59
>2	8 (7.5)	10 (25.6)	0.008
Preterm Labor	6 (5.6)	1 (2.6)	0.68
Gestational HTN	4 (3.7)	2 (5.1)	0.66
PreE without SF	2 (1.9)	0 (0)	1.0
PreE with SF	4 (3.7)	1 (2.6)	1.0
FGR	2 (1.9)	1 (2.6)	1.0
Gestational DM	17 (15.9)	8 (20.1)	0.62
Anemia	38 (35.5)	16 (41)	0.57

Variables presented: LOS = length of stay, PPROM = preterm prelabor rupture of membranes, HTN = hypertension, PreE = pre-eclampsia, SF = severe features, FGR = fetal growth restriction, DM = diabetes mellitus.

**Table 3:** Perioperative factors.

Factor	No GU Injury	GU Injury	*p*-Value
Admission Hgb	10.96 ± 1.3	10.57 ± 1.5	0.23
EBL (mL)	2500 [1800, 4000]	4000 [2500, 7500]	<0.0001
Operative Time (m)	185 [135, 274]	264 [184, 433]	0.0006
UAE	17 (15.9)	10 (25.6)	0.23
Ureteral Stent Placement	50 (46.7)	19 (48.7)	0.85
GU Inury			
Intention Cystotomy	-	11 (28.2)	
Incidental Cystotomy	-	29 (74.4)	
Ureteral	-	5 (12.8)	
ICU Admission	38 (35.5)	26 (66.7)	0.001
ICU LOS	0 [0, 1]	1 [0, 2]	0.0003
Post Op LOS	3 [3, 5]	4 [3, 6]	0.01
Pathology			
Accreta	25 (23.4)	5 (12.8)	0.24
Increta	33 (30.8)	8 (20.5)	0.29
Percreta	49 (45.8)	26 (66.7)	0.04

**Table 4:** Uni-variate and multi-variate analysis.

Factor	OR	95% CI	*p*-Value	aOR	95% CI	*p*-Value
Anterior placentation	4.01	[1.92, 4.67]	0.03	3.73	[1.2, 16.7]	0.04
Percreta by ultrasound	2.89	[1.22, 6.56]	0.01	1.95	[1.1, 6.2]	0.03
Vaginal bleeding > 2	4.27	[1.55, 12.16]	0.005	4.11	[1.4, 12.6]	0.01

## Data Availability

The data presented in this study are available on request from the corresponding author due to patient privacy.
